# Life expectancy of UK physicians in the early 21st century: An analysis of 1,000 fellows from the Royal College of Physicians’ Munk’s Roll

**DOI:** 10.1016/j.clinme.2026.100579

**Published:** 2026-04-06

**Authors:** I. Dafydd Woolley, Peter N. Taylor, Justyna Witczak, Fizzah Iqbal, Anna Scholz, Andrew Lansdown

**Affiliations:** a Cardiff University School of Medicine, Cardiff, UK; b Thyroid Research Group, Systems Immunity Research Institute, Cardiff University School of Medicine, Cardiff, UK; c Department of Immunology and Immunotherapy, School of Infection, Inflammation and Immunology, College of Medicine and Health, University of Birmingham, UK; d Department of Endocrinology, University Hospital of Wales, Heath Park, Cardiff, UK

**Keywords:** Munk’s Roll, Physician mortality, Longevity, Life expectancy, Royal College of Physicians, Gardening, Specialties, Medical workforce, Historical cohort, UK physicians

## Abstract

**Study objective:**

To determine life expectancy among UK physicians using the Royal College of Physicians’ (RCP) Munk’s Roll and to identify demographic or professional factors associated with longevity.

**Design:**

Retrospective observational study of biographical records.

**Setting:**

Munk’s Roll, RCP.

**Participants:**

Random sample of 1,000 fellows from Volume XII (2005–2023).

**Main outcome measures:**

Age at death; associations with sex, birthplace, place of qualification, specialty and hobbies.

**Results:**

995 fellows (915 male, 80 female) were included. Median age at death: 84.0 years (range 34.7–105.0). Men lived longer than women (84.2 vs 80.5 years, p = 0.033). Fellows with obituaries mentioning gardening lived longer than those without (86.0 vs 83.5 years, p = 0.0008). Median age at death varied by region of qualification: North America 94.0, Europe 92.9, Australasia 87.5, Ireland 87.7, UK 84.1, and Indian subcontinent 76.0 years. Age at death differed significantly by region of qualification (p < 0.0001), with fellows qualifying in the Indian subcontinent living notably shorter lives. There were no consistent differences in longevity across specialty groups after adjustment, although pathologists had longer survival (+8.0 years vs cardiologists, p = 0.004).

**Conclusions:**

UK physicians recorded in the Munk’s Roll have substantially higher life expectancy than the general population. Sex differences favouring male physicians and longevity advantages associated with gardening and certain qualification regions were observed. Specialty was not a major determinant of survival. These findings highlight historical inequalities within the profession and the potential importance of wellbeing-related activities in supporting physician longevity.

## Highlights

• UK physicians have substantially higher life expectancy than the general population.
• Longevity advantages were associated with male sex, gardening and certain qualification regions.
• Specialty was not a major determinant of survival.
• Physician wellbeing remains key in supporting longevity and historical inequalities in the medical profession are evident.

## Introduction

Physicians devote their careers to improving the health and wellbeing of others, yet relatively little is known about their own longevity. While the General Medical Council’s *Good Medical Practice*^1^ emphasises the responsibility of doctors to attend to their personal health and wellbeing, the extent to which physicians experience better – or worse – life expectancy than the general population remains incompletely understood. Understanding mortality patterns within the medical profession is not only of historical and sociological interest, but may also inform discussions about occupational stress, working conditions and wellbeing across a medical career.

Several previous studies have examined physician longevity using obituary-based datasets. An analysis of more than 3,000 obituaries published in the *British Medical Journal (BMJ)* between 2003 and 2012 identified associations between life expectancy and factors such as geographic location, career choice and family structure.^2^ A later study of 8,156 *BMJ* obituaries (1997–2019) found that doctors lived longer than the general UK population, with notable variation across specialties, reporting the shortest lifespans among emergency physicians and psychiatrists and the longest among general practitioners and surgeons.^3^ Similar patterns have been reported internationally: a 2024 analysis of Canadian physician obituaries (n > 3,000) again demonstrated longer-than-average lifespans among doctors, with variation across specialties and career pathways.^4^

While physicians generally experience longer lifespans than the general population, emerging evidence suggests that female physicians may not benefit from the same longevity advantages typically observed in women compared to men. A recent landmark study by Patel *et al*^5^ analysed mortality data from over 3.6 million US workers and found that while women had significantly lower mortality rates than men across most occupations, this survival advantage was notably absent among physicians.^5^ The study revealed that female physicians experienced no statistically significant mortality difference compared to male physicians, and for certain causes of death – including cancer and chronic respiratory diseases – female physicians actually had higher mortality rates than their male counterparts. Black female physicians were found to have the highest mortality rates among all physician subgroups.^5^ These findings suggest that systemic workplace factors, including higher rates of burnout, gender discrimination, disproportionate household responsibilities and lower compensation, may be eroding the expected longevity benefits for women in medicine.

Studies also consistently demonstrate that female physicians face unique occupational stressors that may contribute to reduced wellbeing and potentially impact longevity. Women physicians report significantly higher rates of burnout compared to male colleagues and experience more work-related discrimination and microaggressions,^6^ and shoulder disproportionate domestic labour even when married to fellow physicians.^7^ Understanding whether and how these gender-based disparities manifest in mortality patterns among UK physicians is therefore of considerable importance. Despite this growing body of work, there has been no equivalent examination focused specifically on physicians in the UK using a structured, profession-specific biographical database.

The Royal College of Physicians (RCP) Munk’s Roll (now known as Inspiring Physicians) represents a uniquely rich and longstanding archival resource.^8^ First compiled in the 19th century by Dr William Munk, the Roll documents the lives of RCP fellows from the college’s foundation in 1518 to the present day.^8^ Initially published in printed volumes and since 2004 available exclusively online, Munk’s Roll contains detailed biographical entries covering early life, education, medical training, career contributions, personal interests and, where available, dates of birth and death. The service is now dependent on contribution by peers and family members of deceased fellows. Volume XII (2005 onwards) includes nearly 3,000 entries of fellows whose deaths occurred during the early 21st century, making it a high-quality source for contemporary epidemiological and sociocultural analysis. To date, however, the Roll has never been systematically analysed for mortality patterns.

This study therefore aimed to provide the first examination of life expectancy among UK physicians using Munk’s Roll. We analysed a random sample of 1,000 fellows from Volume XII to:

1. Describe life expectancy among UK physicians who died between 2005 and 2023.
2. Compare longevity across sex, birthplace, region of medical qualification and specialty.
3. Explore associations with personal interests.

In doing so, we sought to generate new insights into the demographics, careers and lives of UK physicians, and to contextualise their longevity within existing literature on the medical profession and the wider population.

## Materials and methods

### Study design and data source

This was a retrospective observational study using biographical data from the Royal College of Physicians’ *Munk’s Roll*, a publicly accessible online archive documenting the lives and careers of fellows of the RCP. Volume XII (2005 onwards) was accessed in April 2024.^8^ At the time of extraction, Volume XII contained approximately 3,000 entries of fellows whose deaths had occurred during the early 21st century.

A simple random sample of 1,000 entries was selected from Volume XII. Randomisation was performed using the Microsoft Excel random-number generator applied to the full entry list. This approach ensured unbiased selection irrespective of birth year, specialty or any other characteristic.

The sample size of 1,000 fellows was selected to provide a large and representative subset of entries from Volume XII of Munk’s Roll, which contains approximately 3,000 records. Because each entry contains narrative biographical data rather than structured registry variables, detailed manual extraction was required for each record. A sample of 1,000 therefore represented a pragmatic balance between feasibility and statistical representativeness while still capturing approximately one-third of the available cohort. Random sampling ensured that the analysed group was not influenced by birth cohort, specialty or other characteristics.

For each selected fellow, the following data were extracted manually: sex, date of birth, date of death, age at death (if recorded), place of birth, place of primary medical qualification, specialty (as stated in the obituary), mention of gardening or horticultural interest (binary: Yes/No). Gardening was included because it appeared frequently as a personal interest and was hypothesised to reflect wellbeing-related behaviours.

### Statistical analyses

Entries with: missing dates of birth or death or implausible reconstructed ages (<30 or >110 years) were excluded. After review, 995 fellows remained available for analysis.

Place of medical qualification was categorised into the following regions using predefined geographical groupings: UK, Ireland, North America, Europe (non-UK/Ireland), Australasia, Indian subcontinent, Africa, and Other international. This allowed aggregation of many unique medical schools into statistically meaningful groups. Specialties were grouped into major categories based on recognised clinical domains.

Continuous variables (eg age at death) were summarised using medians and ranges. Categorical variables (eg sex, specialty, region of qualification) were summarised using counts and percentages. Reference categories were selected pragmatically to provide interpretable comparisons: female sex, Africa for region of qualification, and cardiology for specialty group. Age at death was compared between groups using the Mann–Whitney *U* test for two groups or Kruskal–Wallis tests where appropriate due to non-normal distributions. Age at death was summarised primarily using medians rather than means because the distribution of ages was non-normal and included several extreme values. Median-based summaries and non-parametric statistical tests therefore provided more robust estimates that were less sensitive to outliers.

To identify independent predictors of longevity, we fitted a multivariable linear regression model with age at death as the dependent variable. Results are presented as regression coefficients (β) with 95% confidence intervals (CI). Although all fellows in the dataset were deceased (no censoring), Kaplan–Meier survival curves were generated to visually compare the distribution of ages at death between groups by sex and gardening status: A p-value <0.05 was considered statistically significant. All tests were two-sided.

## Results

**Fig. 1:**
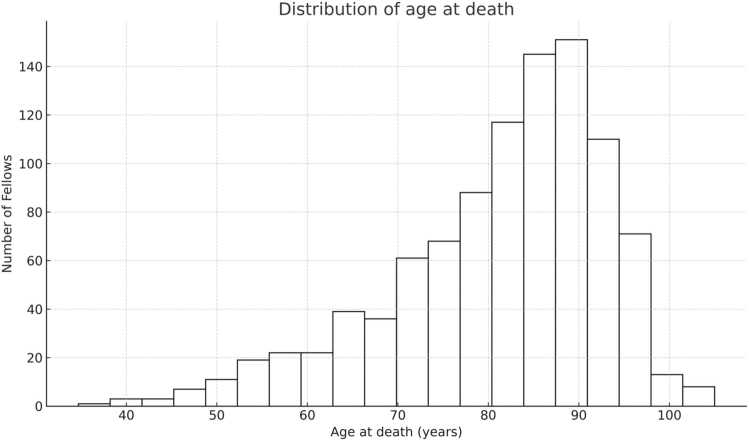
Distribution of age at death among 995 fellows of the RCP.

After excluding entries with irretrievable or implausible age-at-death information, 995 fellows remained for analysis. The cohort consisted of 915 men (91.9%) and 80 women, with a median age at death of 84.0 years (range 34.7–105.0) (Fig. 1). Demographic characteristics are summarised in Table 1.

**Table 1:** Characteristics of the 995 Fellows included in the analysis.

Characteristic	Value
Total fellows analysed	995
**Sex**	
Male	915 (92.0%)
Female	80 (8.0%)
Age at death, median (range)	84.0 (34.7–105.0)
Gardening mentioned	184 (18.5%)
**Region of qualification**	
UK	744 (74.8%)
Ireland	36 (3.6%)
North America	5 (0.5%)
Europe (non-UK/Ireland)	4 (0.4%)
Australasia	54 (5.4%)
Indian subcontinent	39 (3.9%)
Africa	30 (3.0%)
Other international	73 (7.3%)
**Specialty group**	
Cardiology	72 (7.2%)
Respiratory medicine	53 (5.3%)
Endocrinology/diabetes	48 (4.8%)
Gastroenterology	50 (5.0%)
Geriatric medicine	52 (5.2%)
Renal medicine	28 (2.8%)
Paediatrics	123 (12.4%)
Neurology	64 (6.4%)
Dermatology	37 (3.7%)
Psychiatry	33 (3.3%)
Oncology	21 (2.1%)
Haematology	24 (2.4%)
Immunology	18 (1.8%)
Public health	26 (2.6%)
Pathology	24 (2.4%)
General medicine	56 (5.6%)
Other	266 (26.7%)

Male fellows had a higher age at death than female fellows (84.2 vs 80.5 years), p = 0.03. Male sex was associated with greater longevity, with an estimated increase of 3.93 years compared with female sex (95% confidence interval 1.24–6.62; p = 0.004). Gardening remained a strong independent predictor of longer lifespan, associated with an increase of 3.52 years (95% confidence interval 1.64–5.40; p < 0.001).

Age at death varied substantially according to region of qualification. Fellows who qualified in North America (median 94.0 years), Europe (92.9 years), Australasia (87.5 years) and Ireland (87.7 years) tended to live longer than those who qualified in the UK (84.1 years) or Africa (83.6 years). Those who qualified in the Indian subcontinent had notably shorter lifespans compared to the rest of the cohort, with a median age at death of 76.0 years p = 4.3 × 10^−5^ (Table 2). Fellows from North America (p = 0.02) and Australasia (p = 0.0004) had longer life expectancy than those from the UK. Some qualification regions contained relatively small numbers of fellows, particularly North America and Europe, and these comparisons should therefore be interpreted cautiously.

**Table 2:** Multivariable linear regression model of factors associated with age at death.

Predictor	(β), years	95% CI	p-value
Sex: male	3.93	1.24, 6.62	0.004
Gardening mentioned	3.52	1.64, 5.40	<0.001
**Region of qualification**			
Australasia	10.08	5.04, 15.12	<0.001
North America	15.23	4.25, 26.22	0.007
Ireland	6.00	0.35, 11.64	0.037
UK	3.37	−0.89, 7.63	0.12
Europe	5.58	−6.52, 17.69	0.37
Indian subcontinent	−3.89	−9.43, 1.65	0.17
Other international	1.60	−3.35, 6.55	0.53
**Specialty group**			
Pathology	7.96	2.61, 13.30	0.004

Outcome: Age at death (years). Reference groups: female sex, Africa (qualification region), cardiology (specialty).

**Fig. 2:**
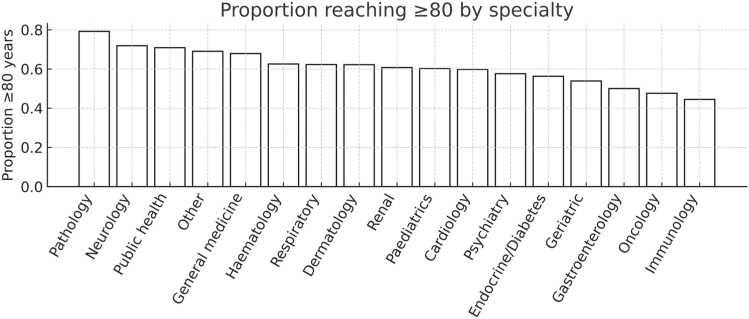
Proportion of fellows surviving to at least 80 years by specialty group.

Differences across specialties were observed descriptively. Pathologists, neurologists and public health physicians had the highest proportion of fellows living to at least 80 years (79%, 72%, and 69%, respectively). Lower proportions were seen in immunology (44%), oncology (48%) and gastroenterology (50%). However, confidence intervals overlapped and these patterns were inconsistent once adjusted for demographic and regional variables. Specialty results are shown in Fig. 2.

Gardening was mentioned as a personal interest in 184 entries (18.5%). Fellows whose obituary mentioned gardening had a higher median age at death than those who did not (86.0 vs 83.5 years), p = 0.008.

Expected cohort effects were seen when analysing year of birth. Those born before 1920 had the highest median ages at death (96.9 years for the 1900–1909 cohort and 92.6 years for the 1910–1919 cohort). Median ages fell for more recent birth cohorts, reflecting incomplete follow-up rather than true declines in longevity.

Few clear differences between specialties were evident after adjustment. Pathologists lived significantly longer than cardiologists in the adjusted model (increase of 7.96 years; p = 0.004), but no other specialty differences remained significant. The overall model explained around 10% of the variation in age at death (R^2^ = 0.095), indicating that additional factors not captured in the obituary likely influence longevity.

## Discussion

This study provides the first systematic analysis of physician life expectancy using the RCP’s Munk’s Roll (now renamed Inspiring Physicians). By examining a contemporary cohort of fellows whose deaths occurred between 2005 and 2023, we have been able to generate novel insights into the demographic, educational and lifestyle factors associated with longevity among UK physicians in the early 21st century. Overall, the median age at death in this cohort was 84.0 years, which appears higher than estimates of life expectancy in the general UK population. For example, national life tables for England and Wales report period life expectancy at birth of approximately 78.6 years for men and 82.6 years for women during the study period.^9^ While these figures are not directly comparable with the age at death of a selected professional cohort, they provide context suggesting that physicians recorded in the Munk's Roll may experience longer lifespans than the general population. Several factors may contribute to this difference, including socioeconomic status, educational attainment, health literacy and access to healthcare. Brayne *et al*^3^ analysed 8,156 doctors’ *BMJ* obituaries from 1997 to 2019, showing that the mean age of death for all specialties was 78.9 years, and specifically for medical specialties was 78.6 years, lower than our study. The reason for the difference in these studies may be that a mean age was recorded rather than median, and the studies were dependent on an obituary being submitted to the *BMJ* and the quality of data included. In contrast, our study examined entries specifically submitted to the RCP Munk’s Roll and were fellows of the RCP. This may reflect physicians of higher socio-economic status and therefore greater life expectancy.

Women in this cohort had lower life expectancy than their male counterparts, a reversal of the pattern seen in the general population. This likely reflects the historical context of the cohort. The female fellows represented in Munk’s Roll Volume XII were predominantly trained in the mid-20th century, a period in which women had limited access to medical education, fewer opportunities for postgraduate advancement and greater workplace discrimination. However, our finding is also consistent with recent evidence from the USA, which demonstrated that female physicians lose the typical female survival advantage observed in other professions.^5^ This is despite women in other occupations – including high-income professions such as law, science and engineering – maintaining substantial longevity advantages over their male counterparts.^5^

Possible explanations include that female physicians often report more burnout symptoms compared to male physicians.^10^ Burnout, characterised by emotional exhaustion and depersonalisation, is associated with cardiovascular disease, depression and substance abuse – all of which could plausibly reduce longevity.^11^ Second, female physicians experience systemic workplace inequities, including gender-based discrimination, lower compensation for equivalent work, and limited advancement opportunities.^6^, ^12^, ^13^ More than 70% of women physicians report experiencing some form of gender discrimination during their careers.^6^ Outside the working environment, female physicians also shoulder disproportionate domestic labour, even when their partners are also physicians. Research demonstrates that in physician couples, women spend significantly more time on household work and childcare than their male partners,^7^ effectively working a ʻsecond shift’ after completing clinical duties. This cumulative burden of professional and domestic responsibilities may contribute to chronic stress and ultimately impact health and longevity.

The current findings from Munk’s Roll, showing reduced female physician longevity, align with these workplace and societal stressors and suggest that the gender disparities documented in contemporary research may have historical precedents and appear deep-rooted in medicine. However, it is important to note that the relatively small number of female fellows in our sample (n = 80) compared to male fellows (n = 915) reflects the historical underrepresentation of women in senior positions within the RCP. Continued monitoring of these patterns in more recent cohorts, as women achieve greater representation in medicine, will be essential to determine whether improving gender equity translates into reduced mortality disparities.

One of the most striking results was the variation in longevity by region of medical qualification. Fellows who qualified in North America and Australasia lived significantly longer than those who trained in the UK, while those from the Indian subcontinent had considerably shorter lifespans. These findings persisted even after adjustment for sex and specialty in multivariable modelling. Differences in longevity by region of qualification may, in part, reflect well-described migration effects. Physicians who trained in North America and Australasia have historically entered UK practice through competitive postgraduate routes or elective career moves, a pattern consistent with the documented healthy migrant effect, in which individuals who relocate internationally tend to be healthier, more socio-economically advantaged, and more mobile than the general population. In contrast, earlier cohorts of doctors from the Indian subcontinent often entered the UK workforce during periods of medical staffing shortages, frequently taking service-intensive posts with fewer opportunities for structured career progression. These longstanding structural differences in migration pathways and professional opportunities may contribute to the divergent longevity patterns observed in our analysis. Small sample sizes in some regions, particularly Europe and North America, mean that these results should be interpreted with appropriate caution, although the statistical evidence in favour of differences for North America and Australasia was strong.

Specialty-related differences in longevity were less pronounced. Although certain specialties, such as pathology, neurology and public health, showed higher proportions of fellows reaching 80 years of age, these differences were not consistent in adjusted analyses. Prior studies, using *BMJ* obituary data, reported shorter lifespans in emergency medicine, anaesthetics, psychiatry and radiology, and longer lifespans in general practice, surgery and public health. The discrepancy likely reflects generational patterns: many fellows in Munk’s Roll Volume XII trained in an era when general internal medicine remained a core part of most hospital physicians’ roles. The distinction between specialties, therefore, may be less reflective of differing exposure profiles than in more modern cohorts.

Although pathology appeared associated with greater longevity in the adjusted model, most specialty comparisons were not statistically significant and confidence intervals overlapped substantially. Differences observed for smaller specialties, such as immunology, should be interpreted cautiously because of limited sample sizes. The discrepancy between these findings and previous studies using BMJ obituary data may also reflect generational differences in the organisation of medical practice, as many physicians in the Munk’s Roll cohort trained during a period when general internal medicine formed a substantial component of most hospital specialties.

A particularly intriguing finding was the association between gardening and increased longevity. Fellows whose biographies mentioned gardening lived, on average, three and a half years longer than those without such mention, and this association persisted after adjustment for sex, specialty and region of qualification. Although no causal inference can be made, gardening may act as a proxy for regular physical activity, time spent outdoors, social engagement, or a balanced lifestyle – factors known to support wellbeing and reduce stress among physicians.^14^ Contemporary literature on physician burnout and mental health reinforces the importance of sustained non-clinical pursuits throughout a medical career. Our findings, while observational, align with the view that wellbeing-supporting activities may contribute to healthier ageing among doctors. Furthermore, this difference may reflect the environment the fellow lived in, with access to a garden aligning with socio-economic, age-related and cultural factors.

Birth cohort effects were also evident, with earlier cohorts demonstrating higher recorded ages at death. This reflects the simple fact that younger cohorts have not yet reached older ages, rather than a true decline in longevity over time. Expanding this work to historical volumes of the Munk’s Roll would allow a more comprehensive assessment of long-term trends in physician mortality across several centuries.

This study has several strengths. It is the first structured interrogation of Munk’s Roll, an unusually rich and longstanding archival resource. The sample size was substantial and randomly selected, reducing selection bias. The availability of personal details, including hobbies, allowed exploration of lifestyle factors seldom captured in traditional mortality datasets.

However, limitations must be acknowledged. Munk’s Roll is not a formal mortality registry, and information within entries varies in detail and accuracy. Cause of death was rarely available, precluding disease-specific analysis. Specialty classification was sometimes ambiguous, reflecting historical practice patterns. The cohort represents fellows, a highly selected group within the medical profession, whose longevity may not reflect that of the wider doctor population. It should also be noted that comparisons with national life expectancy statistics are illustrative rather than strictly equivalent, as life tables represent population-level period estimates whereas the present analysis examines age at death within a historical professional cohort. Finally, although age at death could be reconstructed where missing, a small number of entries contained incomplete timelines or implausible values which required exclusion.

In summary, this analysis demonstrates that UK physicians recorded in the Munk’s Roll enjoy substantially longer life expectancy than the general population. Longevity was associated with sex, region of qualification and, notably, the presence of gardening as a personal interest. The gender disparity in longevity, with female physicians experiencing shorter lifespans than their male colleagues, demands urgent research and attention from medical institutions, regulatory bodies and policymaking organisations. Interventions to address this inequity must target the systemic factors contributing to female physician stress, including implementation of equitable family leave policies, reduction of workplace discrimination, and fair compensation structures.

Specialty differences were limited once adjusted for confounders. These findings provide new insights into the lives and experiences of physicians in the early 21st century and highlight the value of historical biographical archives in understanding health outcomes within the profession. Future work examining earlier volumes of the Munk’s Roll could illuminate long-term trends and changes in physician longevity across the past five centuries.

## CRediT authorship contribution statement

**Peter N. Taylor:** Writing – review & editing, Software, Formal analysis. **Justyna Witczak:** Writing – review & editing, Supervision, Methodology. **I. Dafydd Woolley:** Writing – review & editing, Methodology, Investigation, Data curation. **Fizzah Iqbal:** Writing – review & editing. **Anna Scholz:** Writing – review & editing. **Andrew Lansdown:** Writing – review & editing, Writing – original draft, Resources, Project administration, Methodology, Investigation, Formal analysis.

## Ethics approval and consent to participate

No ethical approval was required for this study.

## Funding

This research did not receive any specific grant from funding agencies in the public, commercial or not-for-profit sectors.

## Declaration of competing interest

The authors declare that they have no known competing financial interests or personal relationships that could have appeared to influence the work reported in this paper.

## Data Availability

Data can be accessed at Munk’s Roll (Inspiring Physicians) at the RCP website: https://history.rcp.ac.uk/inspiring-physicians. Any data analysis can be accessed by contacting the corresponding author.
